# Individual dosimetry system for targeted alpha therapy based on PHITS coupled with microdosimetric kinetic model

**DOI:** 10.1186/s40658-020-00350-7

**Published:** 2021-01-12

**Authors:** Tatsuhiko Sato, Takuya Furuta, Yuwei Liu, Sadahiro Naka, Shushi Nagamori, Yoshikatsu Kanai, Tadashi Watabe

**Affiliations:** 1 Nuclear Science and Engineering Center, Japan Atomic Energy Agency, Shirakata 2-4, Tokai, Ibaraki 319-1195 Japan; 2 Research Center for Nuclear Physics, Osaka University, Suita, Japan; 3 Department of Nuclear Medicine and Tracer Kinetics, Graduate School of Medicine, Osaka University, Suita, Japan; 4 Department of Radiology, Osaka University Hospital, Suita, Japan; 5 Department of Laboratory Medicine, The Jikei University School of Medicine, Tokyo, Japan; 6 Department of Bio-system Pharmacology, Graduate School of Medicine, Osaka University, Suita, Japan

**Keywords:** Individual dosimetry, Targeted alpha therapy, Microdosimetry, EQDX, Monte Carlo

## Abstract

**Background:**

An individual dosimetry system is essential for the evaluation of precise doses in nuclear medicine. The purpose of this study was to develop a system for calculating not only absorbed doses but also EQDX(*α*/*β*) from the PET-CT images of patients for targeted alpha therapy (TAT), considering the dose dependence of the relative biological effectiveness, the dose-rate effect, and the dose heterogeneity.

**Methods:**

A general-purpose Monte Carlo particle transport code PHITS was employed as the dose calculation engine in the system, while the microdosimetric kinetic model was used for converting the absorbed dose to EQDX(*α*/*β*). PHITS input files for describing the geometry and source distribution of a patient are automatically created from PET-CT images, using newly developed modules of the radiotherapy package based on PHITS (RT-PHITS). We examined the performance of the system by calculating several organ doses using the PET-CT images of four healthy volunteers after injecting ^18^F-NKO-035.

**Results:**

The deposition energy map obtained from our system seems to be a blurred image of the corresponding PET data because annihilation γ-rays deposit their energies rather far from the source location. The calculated organ doses agree with the corresponding data obtained from OLINDA 2.0 within 20%, indicating the reliability of our developed system. Test calculations by replacing the labeled radionuclide from ^18^F to ^211^At suggest that large dose heterogeneity in a target volume is expected in TAT, resulting in a significant decrease of EQDX(*α*/*β*) for higher-activity injection.

**Conclusions:**

As an extension of RT-PHITS, an individual dosimetry system for nuclear medicine was developed based on PHITS coupled with the microdosimetric kinetic model. It enables us to predict the therapeutic and side effects of TAT based on the clinical data largely available from conventional external radiotherapy.

## Background

Recently, targeted alpha therapy (TAT) is gaining grounds as a novel treatment for refractory cancer, particularly after an excellent treatment effect of ^225^Ac-PSMA-617 [1]. We have already proved the therapeutic efficacies of [^211^At]NaAt against differentiated thyroid cancer, ^211^At-labeled phenylalanine for glioma, and ^225^Ac-labeled fibroblast activation protein inhibitors (FAPI) against pancreatic cancer in preclinical studies [2–4]. For clinical translation, physicians initiated clinical trial is under preparation using [^211^At]NaAt in patients with differentiated thyroid cancer refractory to radio-iodine (^131^I) treatment. However, the TAT drugs which are successful in clinical application is still limited, and we need practical tools to evaluate the precise dose in the target and risk organs to define the most suitable dose for individual patients.

The absorbed dose (Gy) has generally been used as the primary index for predicting the therapeutic effects on tumor and unintended harmful effects on normal tissue, both in preclinical and clinical trials. In addition, higher relative biological effectiveness (RBE) must be considered in this prediction because α particles densely deposit their energies along their tracks and effectively induce cell killing compared to X-rays and β particles with the same dose. For simplicity, a fixed RBE value of 5 is recommended to use in the dosimetry of TAT [5]. However, actual values of RBE intrinsically depend on the absorbed dose. Thus, explicit consideration of the dose dependence of RBE in the design of TAT is desired in the same way as the carbon ion therapy [6]. In addition, the repair mechanism during the irradiation must also be considered because of a relatively lower dose rate of TAT in comparison to external radiotherapy. Therefore, the concept of the equieffective dose, EQDX(*α*/*β*), formalism was proposed to use in the TAT dosimetry [7], where EQDX represents the absorbed dose to give the same biological effect of the reference treatment, e.g., fractionated X-ray therapy [8]. The commonly used biological effective dose, BED [9], is a special case of EQDX(*α*/*β*). Using EQDX(*α*/*β*), the therapeutic and side effects of TAT can be predicted from the clinical data largely available from conventional external radiotherapy.

Dosimetry systems based on standardized phantoms such as OLINDA/EXM [10] and IDAC-Dose 2.1 [11] are widely used to estimate organ doses in nuclear medicine. However, they have some shortcomings when applied to the targeted radionuclide therapy (TRT) including TAT. For example, they cannot consider detailed anatomical differences of each patient and cannot calculate the heterogeneity of absorbed doses in the target tumor and normal tissues, which may influence tumor response and normal tissue toxicity. Therefore, several authors [12–18] developed 3-dimensional dosimetry systems by automatically creating patient-specific human phantoms and spatial distributions of radionuclides from CT and PET/SPECT images, respectively. These systems allow for a sophisticated design of TRT by calculating more detailed dosimetric quantities such as dose-mass histograms (DMH) in target tumor and normal tissues. In addition, some of them have a function of evaluating BED based on their calculated absorbed doses and dose rates. However, none of the existing system was capable of calculating EQDX(*α*/*β*) for TAT, considering the complex dose dependence of RBE.

Under these situations, we developed a patient-specific dosimetry system that can calculate EQDX(*α*/*β*) for TAT as well as other TRT, based on the Particle and Heavy Ion Transport code System (PHITS) [19] coupled with the microdosimetric kinetic model (MKM) [20]. The accuracy of RBE estimated by PHITS coupled with MKM was well verified for proton therapy [21], carbon-ion therapy [22], and boron neutron capture therapy (BNCT) [23]. In the system, a voxel phantom and a cumulative activity distribution map of a patient are automatically created in the PHITS input format from PET-CT images, respectively. After the PHITS simulation using these input files, EQDX(*α*/*β*) as well as the total absorbed dose and deposition energy in each voxel are estimated, considering the microscopic dose distribution and dose rate. In this study, the performance of the system was examined using the dynamic PET-CT data, and the results were compared with corresponding data obtained from OLINDA 2.0 [10].

## Methods

### Individual dosimetry system based on PHITS

**Fig. 1:**
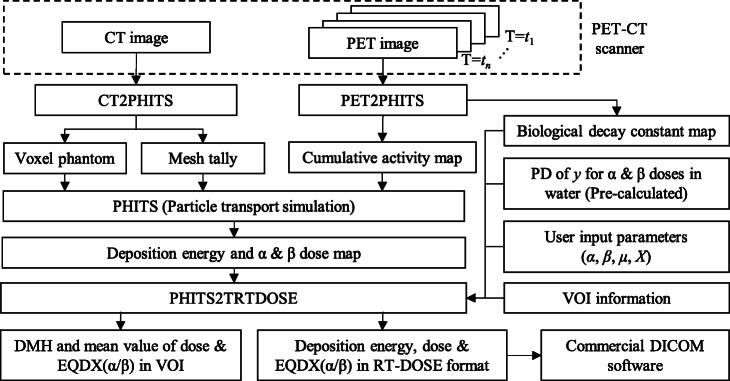
Flowchart of the individual dosimetry system based on PHITS

Figure 1 shows the flowchart of our dosimetry system, which was developed as an extension of the radiotherapy package based on PHITS, so-called RT-PHITS. It can be divided into three processes: (1) conversion from PET-CT images to PHITS input files, (2) calculation of absorbed doses using PHITS, and (3) estimation of EQDX(*α*/*β*) based on the PHITS results coupled with MKM. EQDX(*α*/*β*) as well as total dose and deposition energy in each voxel are converted in DICOM RT-DOSE format. Thus, they can be imported to commercial DICOM software for further analysis. Details of each process are described below.

### Conversion from PET-CT images to PHITS input files

Firstly, the patient-specific voxel phantom in the PHITS input format is created from his/her CT image using the CT2PHITS module, which was formerly called DICOM2PHITS [19]. Then, we adopted the correlation between CT numbers (Hounsfield Unit) and tissue parameters proposed by Schneider et al. [24] in this conversion, though users can define their own formula to represent the correlation in our system. The tallies for scoring the absorbed doses in Gy and deposition energies in MeV are also generated during this process. The resolutions of the created voxel phantom and mesh tallies are the same as the CT image.

A new module of RT-PHITS named PET2PHITS was developed in this study to create the maps of the cumulative activities as well as biological decay constants of the radionuclides based on the PET images. There are two types of patient-specific dosimetry systems; one is to create time-dependent activity maps and execute the particle transport simulations for each time step, and the other is to create a cumulative activity map and execute a single particle transport simulation. Using the former method, dynamical dose evaluation is possible by fitting the calculated doses for each time step. However, it is very time-consuming because the Monte Carlo simulation needs to be continued until sufficiently small statistical uncertainties of the calculated doses in each voxel and time step are obtained to achieve the meaningful fitting. We therefore adopted the latter method; our system determines the cumulative activities and the biological decay constants of the radionuclides by fitting the dynamic PET images. Then, the dose rates are estimated under the assumption that they are proportional to the sum of the physical and biological decay constants of nearby voxels. The detail procedures for determining the cumulative activities and the biological decay constants are shown in Appendix A.

### Calculation of absorbed doses using PHITS

Using the input files created from CT and PET images, PHITS simulation is performed to calculate the absorbed doses in the patient. In this study, PHITS version 3.20 was employed, and the EGS5 mode [25] was used for the photon, electron, and positron transport. The fluences of the source particles including the contributions from daughter nuclides are determined from the RI source generation function in PHITS, based on ICRP Publication 107 [26]. The absorbed doses due to the ionization induced by α and β^±^ particles (referred to α and β doses, respectively) were separately calculated in the simulation. Note that the kerma approximation was not adopted, and thus, the photon doses were categorized as their secondary particle doses, i.e., β dose.

**Fig. 2:**
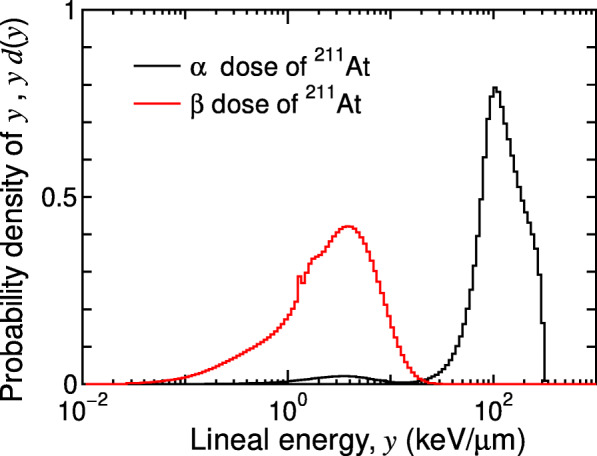
Calculated dose PD of *y*, *yd*(*y*), for *r*_d_ = 0.282 μm for the α and β doses of ^211^At

Before performing the particle transport simulation inside the patient body, another PHITS simulation must be performed to calculate the dose probability densities (PD) of lineal energy, *d*(*y*), in water for α and β doses, which are to be provided to MKM for the RBE estimation. The definition of the fundamental microdosimetric quantities such as lineal energy *y* is described in Appendix B. This simulation is required once for each radionuclide because it is not specific at each patient. The microdosimetric function of PHITS [27] is utilized for this calculation because the site size of *y* needed to be evaluated for MKM is too small (less than 1 μm) to be handled with the condensed history method employed in EGS5. Note that the microdosimetric function was developed by fitting the results of track-structure simulation. Thus, it can analytically determine the PD of *y* down to the nanometer scales, considering the dispersion of deposition energies from the production of δ-rays. Figure 2 shows examples of the calculated PD of *y* for α and β doses of ^211^At.

### Estimation of EQDX(α/β)

EQDX(*α*/*β*) is defined as the total absorbed dose delivered by the reference treatment plan (fraction size *X*) leading to the same biological effect as a test treatment plan [8]. Assuming that the biological effectiveness is proportional to the cell surviving fraction following a linear-quadratic (LQ) relationship, EQDX(*α*/*β*) for a test treatment with the surviving fraction *S* can be calculated by

1 $$ \mathrm{EQDX}\left(\frac{\alpha }{\beta}\right)=\frac{-\ln (S)}{\alpha +\beta X}, $$

where α and β are the LQ parameters for the reference treatment. Based on MKM with the extensions of the saturation correction due to the overkill effect [28] and the dose rate effect [29], the cell surviving fraction in any radiation field with an absorbed dose *D* can be estimated by

2 $$ S(D)=\exp \left[-\left({\alpha}_0+\beta {z}_{1\mathrm{D}}^{\ast}\right)D- G\beta {D}^2\right], $$

where *α*_0_ is the linear coefficient of the surviving fraction with the limit of LET → 0, *G* is the correction factor due to the dose rate effect, and ${z}_{1\mathrm{D}}^{\ast }$ is the saturation-corrected dose-mean specific energy, deduced by

3 $$ {z}_{1\mathrm{D}}^{\ast }=\frac{1}{\pi {r}_{\mathrm{d}}^2}{y}^{\ast }=\frac{1}{\pi {r}_{\mathrm{d}}^2}{y}_0^2\int \frac{\left[1-\exp \left(-{y}^2/{y}_0^2\right)\right]d(y)}{y}\mathrm{d}y, $$

where y^*^ is the saturation-corrected lineal energy, *r*_d_ is the radius of a subcellular structure referred to as domain, *y*_0_ is a so-called saturation parameter that indicates the lineal energy above which the saturation correction due to the overkill effect becomes very important, and *d*(*y*) is the dose probability density in domain. *d*(*y*) in each voxel can be determined from its α and β doses, *D*_α_ and *D*_β_, respectively, as written by

4 $$ d(y)=\frac{D_{\alpha }{d}_{\alpha }(y)+{D}_{\beta }{d}_{\beta }(y)}{D_{\alpha }+{D}_{\beta }}, $$

where *d*_α_(*y*) and *d*_β_(*y*) are their dose PD for each radionuclide precalculated by PHITS using the microdosimetric function. More detailed descriptions about the features of MKM are given in Appendix B in addition to the definition of fundamental microdosimetric quantities.

Assuming that the dose rates of TRT are expressed as a mono-exponential function with a decay constant of λ_phy_ + λ_bio_, where λ_phy_ and λ_bio_ are the physical and biological decay constants, respectively, the value of *G* can be calculated using [13]

5 $$ G=\frac{\lambda_{\mathrm{phy}}+{\lambda}_{\mathrm{bio}}}{\mu +{\lambda}_{\mathrm{phy}}+{\lambda}_{\mathrm{bio}}}, $$

where *μ* is the recovery rate constant. The parameters *α*, *β*, *μ*, *α*_0_, *r*_d_, and *y*_0_ depend on the cell line. Among them, *α*_0_, *r*_d_, and *y*_0_ are specific to MKM, and their determination requires the experimental data of cell surviving fractions for various ion irradiations, which are generally not available. Thus, we fixed *r*_d_ and *y*_0_ to 0.282 μm and 93.4 keV/μm, respectively, which were evaluated from the surviving fractions of the HSG cell irradiated by various radiations including He ions [30, 31], and calculated *α*_0_ from *α* and ${z}_{1\mathrm{D}}^{\ast }$ for the reference radiation, ${z}_{1\mathrm{D},\mathrm{ref}}^{\ast }$, using the equation of ${\alpha}_0=\alpha -\beta {z}_{1\mathrm{D},\mathrm{ref}}^{\ast }$. Then, the user input parameters to our dosimetry system are *α*, *β*, and *μ*, which can be obtained from the measured surviving fractions of the reference radiation, as well as the fraction size *X*. Referring to our previous works [23, 30], we set *α* = 0.251 Gy^−1^, *β* = 0.0615 Gy^−2^, *μ* = 1.5 h^−1^, and *X* =2 Gy in the test simulations performed in this study. Consequently, EQDX(*α*/*β*) calculated in this study can be expressed as EQD2(4.08), where 4.08 is the *α*/*β* ratio, i.e., 0.251/0.0615.

EQDX(*α*/*β*) in a certain voxel can be simply calculated from Eq. 1 by substituting the surviving fraction in the voxel obtained from Eq. 2. In contrast, special care should be taken when EQDX(*α*/*β*) in a certain volume of interest (VOI) consisting of multiple voxels such as tumor and normal tissue is calculated because of the non-linear relationship between the EQDX(*α*/*β*) and the surviving fraction. In such cases, the mean surviving fraction in VOI, *S*_VOI_, is given by

6 $$ {S}_{\mathrm{VOI}}=\frac{\sum_i{S}_i\left({D}_i\right){m}_i}{\sum_i{m}_i}, $$

where *S*_*i*_, *D*_*i*_, and *m*_*i*_ are the surviving fraction, dose, and mass, respectively, of voxel *i* made up of VOI. EQDX(*α*/*β*) in VOI can be obtained from Eq. 1 by supplying *S*_VOI_ to *S* in similar to the concept of the equivalent uniform dose (EUD) [32]. DMH in VOI is the key quantity in this evaluation, which can be also calculated from our dosimetry system. These calculations are performed by a newly developed module of RT-PHITS named PHITS2TRTDOSE.

### Dynamic PET-CT acquisition and analysis

This study was approved by the institutional review board, and written informed consents were obtained from all participants. The performance of the system was examined using the dynamic PET-CT data of four healthy volunteers after injecting ^18^F-labeled NKO-035 with 221.6 ± 3.8 MBq, which is a specific substrate of L-type amino acid transporter-1 (LAT1). The dynamic PET data were acquired in nine frames (total scan duration: 90 min) with low-dose CT scan. All images were depicted by OSIRIX (Newton Graphics, Inc., Sapporo, Japan). Details of the data acquisition procedures were described in Appendix C. In the dose estimation, NKO-035 was assumed to be labeled with not only ^18^F but also ^211^At, ^131^I, and ^177^Lu with the same distribution in the body. Volume of interest was placed in major organs on dynamic PET images using PMOD software (PMOD Technologies Ltd., Zurich, Switzerland) with reference to CT images. The residence times in major organs and tissues were estimated for each patient based on their dynamic PET data using the method described in Appendix A. Supplying those data into OLINDA 2.0, the organ doses were calculated and compared with the corresponding data obtained from our dosimetry system by Bland-Altman analysis.

## Results

**Fig. 3:**
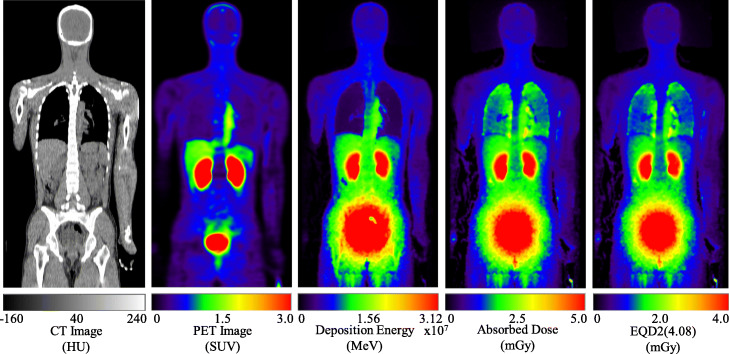
Representative CT and PET images (90 min average) of a volunteer after the injection of NKO-035 labeled with ^18^F and the corresponding deposition energy, absorbed dose, and EQD2(4.08) maps obtained from RT-PHITS

Figure 3 shows the coronal view of CT and PET scans for a volunteer after injecting ^18^F-NKO-035, and the corresponding deposition energy, absorbed dose, and EQD2(4.08) maps, where 4.08 is the *α*/*β* ratio. The history number of the PHITS simulation was set to 300 million so that the statistical uncertainties are very small. The deposition energy map seems to be a blurred image of the PET data particularly around the high-activity organs such as kidney and bladder because annihilation γ-rays deposit their energies rather far from the source location. In contrast, the dose and EQD2(4.08) maps exhibit higher values even at low activity regions such as the lungs. This is because the dose and EQDX(*α*/*β*) are closely related to the activity per mass (and not volume) and consequently tend to be higher at low-density regions. The relative distributions of the dose and EQD2(4.08) are similar to each other, though the absolute values of EQD2(4.08) are approximately 74% of the corresponding dose as discussed later.

**Fig. 4:**
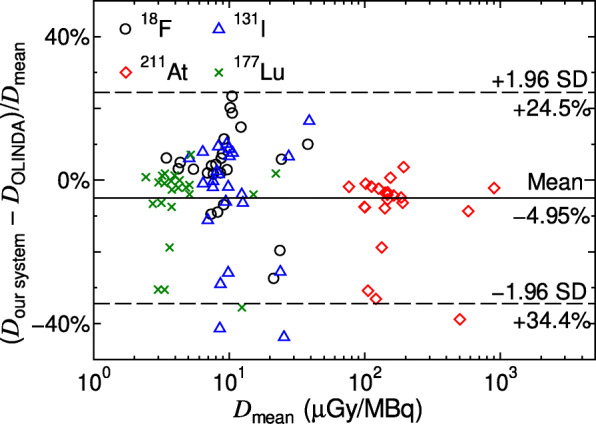
Bland-Altman plot between the mean and percent difference of the organ doses calculated by RT-PHITS and OLINDA 2.0 for each volunteer, radioisotope, and organ. The percent differences were obtained from (*D*_RT-PHITS_ − *D*_OLINDA_)/*D*_mean_, where *D*_RT-PHITS_ and *D*_OLINDA_ are organ doses calculated by RT-PHITS and OLINDA 2.0, respectively, while *D*_mean_ is the mean value of the two doses

Table 1 summarises the absorbed doses in the brain, lung, liver, spleen, pancreas, and kidney obtained from RT-PHITS and OLINDA 2.0. It constitutes the mean values and standard deviations of the four volunteers after injection of NKO-035 virtually labeled with 1 MBq of ^18^F, ^211^At, ^131^I, or ^177^Lu. The mean organ doses for four volunteers calculated by RT-PHITS agree with the corresponding OLINDA data mostly within 20%. Figure 4 shows the Bland-Altman plot between the mean and percent difference of the organ doses calculated by RT-PHTIS and OLINDA 2.0 for each volunteer, radioisotope, and organ. It is evident that data are scattered randomly with respect to the mean organ doses.

**Table 1:** Absorbed doses (μGy) in the brain, lung, liver, spleen, pancreas, and kidney obtained from RT-PHITS and OLINDA 2.0. The data are the mean value and standard deviation (S.D.) of the four volunteers with the injection of NKO-035 virtually labeled with 1 MBq of ^18^F, ^211^At, ^131^I, or ^177^Lu

	^18^F				^211^At				^131^I				^177^Lu			
	RT-PHITS		OLINDA		RT-PHITS		OLINDA		RT-PHITS		OLINDA		RT-PHITS		OLINDA	
	Mean	S.D.	Mean	S.D.	Mean	S.D.	Mean	S.D.	Mean	S.D.	Mean	S.D.	Mean	S.D.	Mean	S.D.
Brain	4.49	0.83	4.31	0.85	104	28.8	108	30.6	7.15	2.22	6.81	2.06	3.44	1.12	3.50	1.15
Lung	8.36	0.82	8.06	0.68	170	23.5	174	19.3	9.20	1.49	9.02	1.24	4.51	0.72	4.49	0.55
Liver	9.40	0.50	9.10	0.53	127	6.07	137	10.3	8.67	0.40	9.20	1.52	3.40	0.15	3.64	0.34
Spleen	7.56	0.60	7.80	0.72	99.4	8.92	120	16.0	7.06	0.45	8.80	1.50	2.75	0.21	3.28	0.46
Pancreas	12.0	0.86	9.90	1.01	156	20.3	161	23.7	11.2	1.10	11.07	2.02	4.25	0.55	4.29	0.64
Kidney	26.4	9.67	27.7	5.94	547	246	673	158	27.8	10.3	30.1	4.31	14.0	6.15	16.7	3.52

## Discussion

We have developed an individual dosimetry system, including the function for calculating EQDX(*α*/*β*), based on PHITS coupled with the microdosimetric kinetic model. The agreements between the calculated doses obtained from RT-PHITS and OLINDA 2.0 are quite satisfactorily, confirming the reliability of our developed system. In addition, no apparent trend is observed in the Bland-Altman plot drawn in Fig. 4, suggesting that the discrepancies between RT-PHITS and OLINDA results are predominantly attributed to random issues such as anatomical differences between each volunteer and the standardized phantom adopted in OLINDA 2.0. For example, data with the percent difference out of ± 1.96 S.D. are for organs whose masses differ from those of the standardized phantom by more than 30%.

**Fig. 5:**
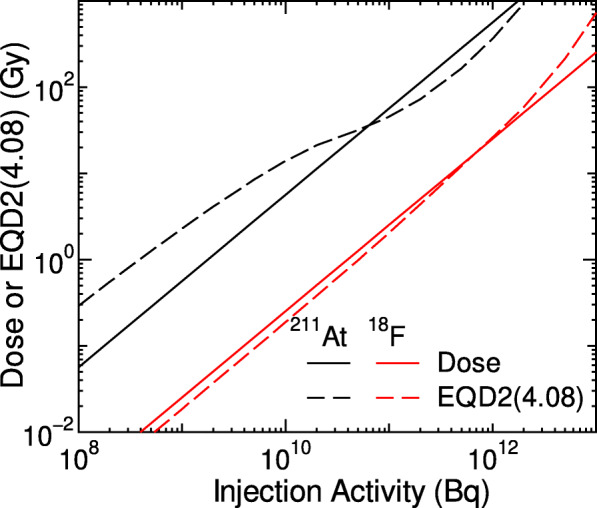
Activity dependences of the calculated dose and EQD2(4.08) in the kidney for volunteer 1 with the injection of NKO-035 labeled with ^211^At or ^18^F

Figure 5 shows the activity dependency of the calculated dose and EQD2(4.08) in the kidney for a volunteer after injecting NKO-035 labeled with ^211^At or ^18^F. The calculated doses are directly proportional to the injection activity because the biokinetics of the radionuclides are assumed to be independent of their activity in this calculation. In contrast, EQD2(4.08) complicatedly depend on the injection activity. For ^211^At, they are higher and lower than the corresponding dose at lower and higher activities, respectively, and vice versa for ^18^F.

**Fig. 6:**
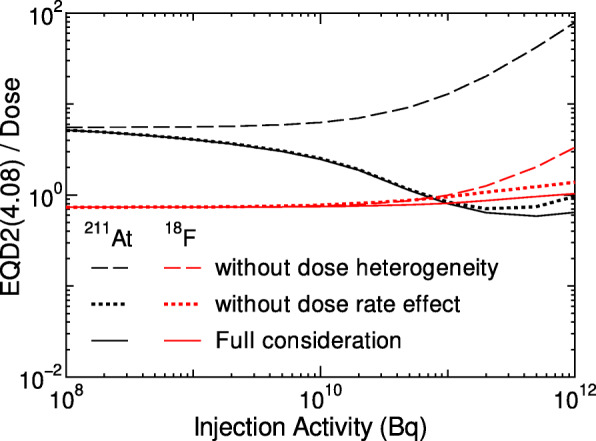
Activity dependences of the ratios of EQD2(4.08) to the corresponding dose for the kidney. The data for EQD2(4.08) calculated without considering the dose heterogeneity or the dose-rate effect are also plotted

**Fig. 7:**
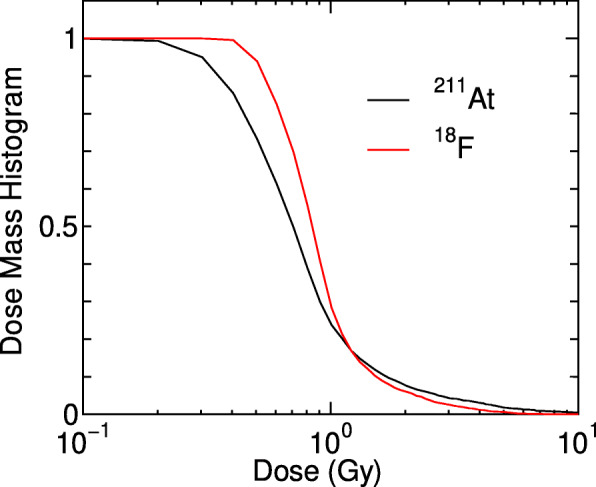
Dose mass histogram (DMH) in the kidney for volunteer 1 with the injection of NKO-035 labeled with ^211^At or ^18^F. The mean doses were adjusted to 1 Gy for both DMH

In order to clarify these complicated relationships, we calculated EQD2(4.08) without considering the dose heterogeneity by simply averaging EQD2(4.08) in the kidney, and those without considering the dose-rate effect by setting the recovery rate constant *μ* = 0. Figure 6 shows the ratios of each EQD2(4.08) to the corresponding absorbed dose as a function of the injection activity. It is evident from the graph that ignoring the dose heterogeneity results in the increase of EQD2(4.08) particularly when injecting ^211^At with higher activities. This tendency can be explained due to the following. Firstly, the surviving fractions at high-dose irradiation are predominantly determined from those of cells having relatively smaller doses as discussed in our previous paper [33]. Lastly, the dose heterogeneity is relatively large for the injection of ^211^At in comparison to ^18^F, as shown in Fig. 7. Therefore, the consideration of the dose heterogeneity in a target volume is indispensable in the clinical design of TAT. The ignorance of the dose-rate effect also results in the increase of EQD2(4.08), but its influence is not so significant and is limited only at higher activities. This is because the dose rates are not very low in the studied cases owing to rather short half-lives of ^211^At and ^18^F, and the dose-rate effect reduces the coefficient of the quadratic term as expressed in Eq. 2, which is important only at high-dose irradiation. Note that the ratio of EQD2(4.08) to dose at lower activities becomes closer to 5.5 and 0.74 for ^211^At and ^18^F, respectively, which correspond to RBE at the limit of *D* → 0, RBE_M_, multiplied with *α*/(*α*+*βX*).

It should be mentioned that the model parameters used in these test calculations were determined from the surviving fractions of cells irradiated with external radiations, which might be inappropriate to be used for representing the surviving fraction of TAT because the absorbed doses are heterogeneously distributed in a microscopic scale due to the heterogeneity of radionuclides among each cell compartment [34] and organ microstructure [35]. Thus, the evaluation of the reliable model parameters is the key issue for introducing RT-PHITS in the preclinical study of TAT. For precisely calculating the doses in organs with fine structure such as stomach wall, implementation of tetrahedral-mesh phantoms in RT-PHITS is ongoing by introducing the technology developed by another PHITS-based internal dosimetry tool PARaDIM [36]. Reduction of the computational time is also desirable before the practical use of RT-PHITS in the clinic because PET-CT data of patients are generally confidential and not able to be transferred to a high-performance computer that is publicly accessible. Currently, the conventional organ dose calculation using RT-PHITS costs less than a few CPU hours, but the precise estimation of EQDX requires at least 100 CPU hours because the statistical uncertainties in each voxel must be very small in the calculation.

## Conclusion

As an extension of RT-PHITS, we developed an individual dosimetry system dedicated to nuclear medicine particularly to TAT based on PHITS coupled with the microdosimetric kinetic model. It calculates not only absorbed doses but also EQDX(*α*/*β*) from the PET-CT images, considering the dose dependence of RBE, the dose-rate effect, and the dose heterogeneity. With these functionalities, RT-PHITS enables us to predict the therapeutic and side effects of TAT based on the clinical data largely available from conventional external radiotherapy. RT-PHITS including the modules developed in this study has been implemented in the latest version of PHITS, which is freely available upon the request to Japan Atomic Energy Agency.

## Figures and Tables

**Fig. 8:**
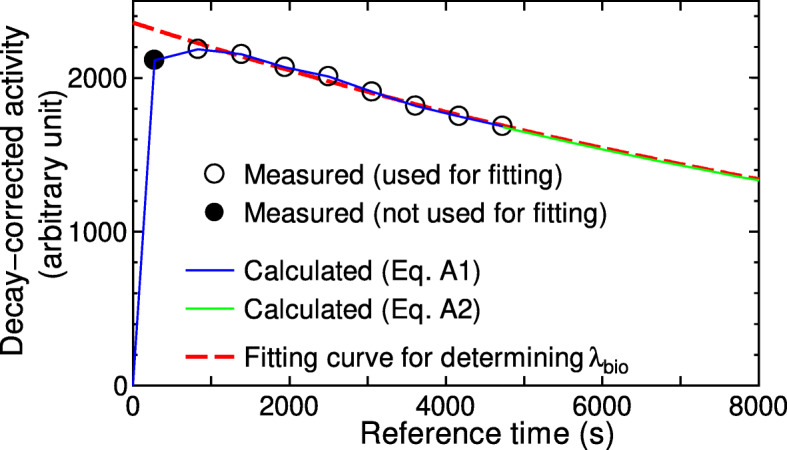
Example of the decay-corrected activities calculated from Eqs. 7 and 8 in comparison with the measured data. The fitting curve for determining λ_bio_ is also depicted

**Fig. 9:**
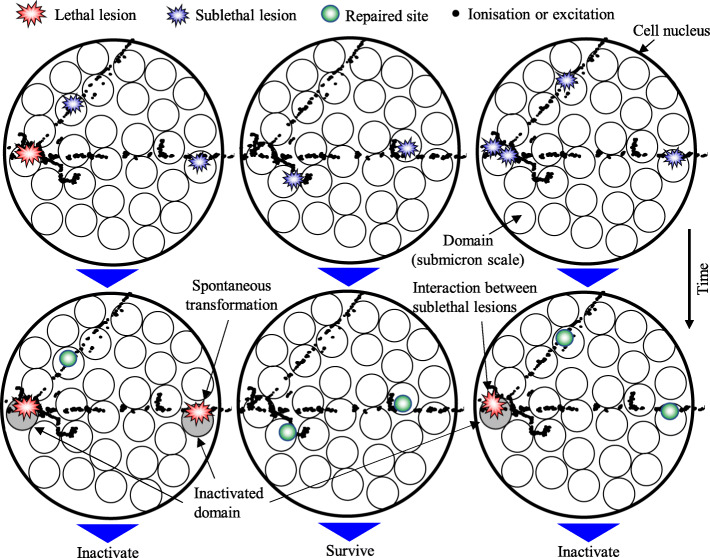
Schematic drawing of the concept of MKM

## Data Availability

The datasets used and/or analyzed during the current study are available from the corresponding author on reasonable request.

## Appendix A

### Procedure for determining the cumulative activities and the biological decay constants

In general, the cumulative activities are estimated by integrating the time-activity curve determined from a mono-exponential or bi-exponential fitting of the PET/SPECT images [37]. However, such fitting procedures are occasionally failed particularly when the statistical fluctuation of the measured activities was high or the number of the time steps of PET/SPECT images was small. We therefore employed a simple method for determining the decay-corrected activities, *A*(*t*), by linearly interpolating the corresponding data obtained from the *i*th measurement of PET/SPECT, *A*_*i*_, and by extrapolating the last data, *A*_*n*_, under the assumption of the mono-exponential decay, as written below:

7 $$ A(t)={a}_it+{b}_i=\frac{A_i-{A}_{i-1}}{t_i-{t}_{i-1}}t+\frac{A_{i-1}{t}_i-{A}_i{t}_{i-1}}{t_i-{t}_{i-1}}\kern1em \mathrm{for}\kern0.75em {t}_{i-1}<t\le {t}_i\kern0.5em \left(i=1\dots n\right) $$

8 $$ A(t)={A}_n{e}^{-{\lambda}_{\mathrm{bio}}\left(t-{t}_n\right)}\kern0.75em \mathrm{for}\kern1em t>{t}_n, $$

where *t*_*i*_ is the reference time of the *i*^th^ measurement, and λ_bio_ represents the decay constant of the radiopharmaceutical due to the biological clearance. Note that we assumed *A*_0_ = *t*_0_ = 0 in this calculation. The numerical value of λ_bio_ is determined from the least-square fitting of *A*_*i*_ by the mono-exponential function, excluding the data before the peak or below a certain threshold value. The fitting is regarded to be failed in the case that the fitted decay constant is negative. The actual value of λ_bio_ used in Eq. 8 as well as Eq. 5 is calculated by averaging the fitted decay constants of 10 nearby voxels where the fitting was succeeded. Figure 8 shows an example of the decay-corrected activities obtained from Eqs. 7 and 8 in comparison with the measured data.

The cumulative activity, *C*, can be mathematically derived from *A*(*t*) as follows:

9 $$ {\displaystyle \begin{array}{c}C=\frac{\lambda_{\mathrm{phy}}}{\lambda_{\mathrm{phy},\mathrm{PET}}}{\int}_0^{\infty }A(t){e}^{-{\lambda}_{\mathrm{phy}}t}\mathrm{d}t\\ {}=\frac{\lambda_{\mathrm{phy}}}{\lambda_{\mathrm{phy},\mathrm{PET}}}\left[\sum \limits_{i=1}^n{\int}_{t_{i-1}}^{t_i}\left({a}_it+{b}_i\right){e}^{-{\lambda}_{\mathrm{phy}}t}\mathrm{d}t+{A}_n{e}^{-{\lambda}_{\mathrm{phy}}{t}_n}{\int}_{t_n}^{\infty }{e}^{-\left({\lambda}_{\mathrm{phy}}+{\lambda}_{\mathrm{bio}}\right)\left(t-{t}_n\right)}\mathrm{d}t\right]\\ {}=\frac{\lambda_{\mathrm{phy}}}{\lambda_{\mathrm{phy},\mathrm{PET}}}\left[\sum \limits_{i=1}^n\frac{\left({\lambda}_{\mathrm{phy}}{t}_{i-1}+1\right){e}^{-{\lambda}_{\mathrm{phy}}{t}_{i-1}}-\left({\lambda}_{\mathrm{phy}}{t}_i+1\right){e}^{-{\lambda}_{\mathrm{phy}}{t}_i}}{\lambda_{\mathrm{phy}}^2}{a}_i+\frac{e^{-{\lambda}_{\mathrm{phy}}{t}_{i-1}}-{e}^{-{\lambda}_{\mathrm{phy}}{t}_i}}{\lambda_{\mathrm{phy}}}{b}_i+\frac{A_n{e}^{-{\lambda}_{\mathrm{phy}}{t}_n}}{\left({\lambda}_{\mathrm{phy}}+{\lambda}_{\mathrm{bio}}\right)}\right].\kern23.75em \end{array}} $$

where λ_phy_ and λ_phy,PET_ is the physical decay constants of the radionuclides used for TRT and PET/SPECT, respectively.

## Appendix B

### Definition of fundamental microdosimetric quantities and basic features of MKM

The most important feature of microdosimetry in comparison to the conventional-scale dosimetry is the consideration of the spatial and stochastic divergences of deposition energies around the trajectories of charged particles. Thus, two stochastic quantities specially used in microdosimetry, i.e., specific energy *z* in Gy and lineal energy *y* in keV/μm, were defined and measured instead of the corresponding non-stochastic quantities used in conventional dosimetry, i.e. absorbed dose and linear energy transfer (LET), respectively. The definitions *z* and *y* are as follows:

10 $$ z=\frac{\varepsilon }{m}, $$

11 $$ y=\frac{\varepsilon }{\overline{l}}, $$

where *ɛ* is the energy imparted to a target with mass *m* and mean chord length $\overline{l}$. They are generally expressed in their frequency or dose probability density functions, *f*(*z*) and *f*(*y*) or *d*(*z*) and *d*(*y*), respectively. Detailed descriptions on the definition of the microdosimetric quantities are given in International Commission on Radiation Units and Measurements (ICRU) Report 36 [38].

MKM [20] is one of the most successful models to explain the biological effectiveness for the cellular surviving fraction. It mathematically interprets the LQ relation of the surviving fraction based on the theory of dual radiation action [39]. The concept of MKM is schematically drawn in Fig. 9. In MKM, the following six basic assumptions were made: (i) a cell nucleus can be divided into multiple domains with submicron scales; (ii) radiation exposure produces two types of DNA damage named lethal and sublethal lesions in cell nuclei; (iii) the number of lethal and sublethal lesions produced in a domain is proportional to the specific energy, *z*, in the domain; (iv) a sublethal lesion is to be repaired, or converted into a lethal lesion via spontaneous transformation or interaction with another sublethal lesion created in the same domain; (v) a domain is to be considered inactivated when an intra-domain lethal lesion is formed; and (vi) a cell is to be considered inactivated when an intranuclear domain is inactivated.

Based on these assumptions, it can be mathematically derived that the cell surviving fraction in any radiation field with an absorbed dose *D*, *S*_MK_(*D*), is calculated by

12 $$ {S}_{\mathrm{MK}}(D)=\exp \left[-\left({\alpha}_0+\beta {\overline{z}}_{1\mathrm{D}}\right)D-\beta {D}^2\right], $$

where *α*_0_ and *β* are the parameters independent of the radiation field, and ${\overline{z}}_{1\mathrm{D}}$ denotes the dose-mean specific energy per event in domain. Considering the saturation correction due to the overkill effect [28] and the dose rate effect [29], Eq. 12 can be replaced by Eq. 2.

The most important feature of MKM in comparison to other conventional LQ models is that it can computationally determine the α parameter for any radiation field considering the spatial and stochastic divergences of the deposition energies, owing to the use of the microdosimetric quantities *z* and *y* instead of LET. Thus, the RBE values of low-energy He ions calculated by MKM are higher than the corresponding data for high-energy C ions having the same LET, as expected from the track structure simulation and experimental data [31]. In addition, MKM suggests that the *β* parameter should be independent of the radiation field, while conventional LQ models regard it as a variable and generally presume the value to be 0 for high-LET radiation. Thus, RBE obtained from by MKM are always greater than 1 in contrast to those calculated by conventional LQ models, which are occasionally less than 1 in the case of high-dose and high-LET irradiations with *β* = 0. These features are beneficial in the estimate of RBE for TAT.

## Appendix C

### Procedure for PET-CT data acquisition

Whole body PET/CT images were acquired using SET-3000BCT/X, (SHIMADZU, Kyoto, Japan) in 3-D mode (pixel size 4.0 mm, slice thickness 3.25 mm) with 9 min per frame (from the mid-thigh to top-skull). PET images were reconstructed by Dynamic Row-Action Maximum Likelihood Algorithm (DRAMA) with an image matrix of 128 × 128, and a voxel size of 4.0 × 4.0 × 3.25 mm^3^. Attenuation correction was performed using ^137^Cs source. The unenhanced low-dose CT was acquired after PET scan (120 kVp and 37.5 mAs). The CT-scans were reconstructed to a slice thickness of 5 mm.

## Abbreviations

BED: Biological effective dose
DMH: Dose-mass histgram
EQD: Equieffective dose
EUD: Equivalent uniform dose
LET: Linear energy transfer
MKM: Microdosimetric kinetic model
PD: Probability density
PHITS: Particle and Heavy Ion Transport code System
RBE: Relative biological effectiveness
RT-PHITS: Radiotherapy package based on PHITS
TAT: Targeted alpha therapy
TRT: Targeted radionuclide therapy
VOI: Volume of interest
